# Radiation therapy for *de novo* anorectal cancer in patients with a history of prostate radiation therapy

**DOI:** 10.3389/fonc.2022.975519

**Published:** 2022-09-15

**Authors:** Lara Hilal, Abraham J. Wu, Marsha Reyngold, John J. Cuaron, John Navilio, Paul B. Romesser, Alexandra Dreyfuss, Sean Yin, Zhigang Zhang, Xing Bai, Sean L. Berry, Melissa Zinovoy, Maliha Nusrat, Emmanouil Pappou, Michael J. Zelefsky, Christopher H. Crane, Carla Hajj

**Affiliations:** ^1^ Department of Radiation Oncology, American University of Beirut Medical Center, Beirut, Lebanon; ^2^ Department of Radiation Oncology, Memorial Sloan Kettering Cancer Center, New York, NY, United States; ^3^ Department of Medical Physics, Memorial Sloan Kettering Cancer Center, New York, NY, United States; ^4^ Department of Epidemiology and Biostatistics, Memorial Sloan Kettering Cancer Center, New York, NY, United States; ^5^ Gastrointestinal Oncology Service, Memorial Sloan Kettering Cancer Center, New York, NY, United States; ^6^ Department of Surgery, Memorial Sloan Kettering Cancer Center, New York, NY, United States

**Keywords:** prostate radiation therapy, anorectal cancer, second course of pelvic RT, toxicity, fistula

## Abstract

**Introduction:**

Radiation therapy (RT) for anorectal cancer after prior prostate cancer RT is usually avoided due to concern for complications. Data on this topic is scarce. Our aim was to evaluate tolerability, toxicity, and clinical outcomes associated with a second course of pelvic radiation in men with *de novo* anorectal cancers previously treated with RT for prostate cancer.

**Materials/methods:**

We conducted a single-institution retrospective study of men treated with RT for rectal or anal cancer after prior prostate RT. Toxicity data were collected. Treatment plans were extracted to assess doses to organs at risk and target coverage. Cumulative incidence was calculated for local and distant progression. Kaplan-Meier curves were used to estimate overall survival (OS) and progression-free survival (PFS).

**Results:**

We identified 26 patients who received anorectal RT after prostate cancer RT: 17 for rectal cancer and 9 for anal cancer. None had metastatic disease. Prior prostate RT was delivered using low dose rate brachytherapy (LDR), external beam RT (EBRT), or EBRT + LDR. RT for rectal cancer was delivered most commonly using 50.4Gy/28 fractions (fr) or 1.5 Gy twice-daily to 30-45 Gy. The most used RT dose for anal cancer was 50Gy/25 fr. Median interval between prostate and anorectal RT was 12.3 years (range:0.5 - 25.3). 65% and 89% of rectal and anal cancer patients received concurrent chemotherapy, respectively. There were no reported ≥Grade 4 acute toxicities. Two patients developed fistulae; one was urinary-cutaneous after prostate LDR and 45Gy/25fr for rectal cancer, and the other was recto-vesicular after prostate LDR and 50Gy/25fr for anal cancer. In 11 patients with available dosimetry, coverage for anorectal cancers was adequate. With a median follow up of 84.4 months, 5-yr local progression and OS were 30% and 31% for rectal cancer, and 35% and 49% for anal cancer patients, respectively.

**Conclusion:**

RT for anorectal cancer after prior prostate cancer RT is feasible but should be delivered with caution since it poses a risk of fistulae and possibly bleeding, especially in patients treated with prior LDR brachytherapy. Further studies, perhaps using proton therapy and/or rectal hydrogel spacers, are needed to further decrease toxicity and improve outcomes.

## Introduction

Prostate cancer treatment with radiation therapy (RT) is associated with an increased risk of developing secondary malignancies, mainly rectal cancer. In a study from the SEER database on around 30,550 men with prostate cancer who received RT, RT was independently associated with the development of rectal cancer over time (1). The standard treatment of locally advanced rectal cancer (LARC) includes radiation therapy, chemotherapy and surgery (2). The treatment of LARC after previous pelvic irradiation must balance concerns for toxicity with the improvement in local control and the possibility for organ preservation. Another challenging clinical scenario is the treatment of anal cancer in patients with a history of prostate RT. Chemoradiation therapy is the standard curative treatment for anal cancer and can improve patients’ colostomy-free survival and quality of life (3).

Reirradiation for rectal cancer has been reported for recurrent rectal tumors using a hyperfractionated regimen of 1.5 Gy twice daily, with a median total dose of 39 Gy. Grades 3-4 late toxicities of this regimen were reported to be around 34% and included small bowel obstruction, urinary obstruction and fistula formation (4). The same regimen of hyperfractionated 1.5 Gy twice daily has been used to treat 10 patients with locally recurrent anal squamous cell carcinoma in a study by Osborne et al. They reported 3-year disease-free survival of 40% and 3-year overall survival of 60% with one grade 3 acute toxicity and no grade 3 or higher late toxicity (5).

However, data is scarce on *de novo* rectal or anal cancer radiation therapy after prior radiotherapy for prostate cancer. In a paper from Stanford Cancer Institute, Massachusetts General Hospital (MGH), and MD Anderson Cancer Center (MDACC), radiation oncology experts were posed a question on delivering a second course of radiation therapy for rectal cancer 2 years after radiation therapy for prostate cancer. The consensus among them was that this scenario presents a clinical challenge and that non-radiotherapy options are preferred whenever feasible. In cases where RT is necessary, bladder toxicity would be a concern and limiting overlap with previously irradiated adjacent organs at risk is essential (6).

The aim of our study was to evaluate outcomes and toxicity associated with RT for do novo anorectal cancer in patents previously treated with prostate RT.

## Materials and methods

We conducted a single-institution retrospective study that included patients treated by radiation for rectal or anal cancer after prior RT for prostate cancer.

This study has been approved by Memorial Sloan Kettering Cancer Center Institutional Review board (MSKCC IRB 16- 370) and individual consent for this retrospective analysis was waived.

From our institutional database, we identified all patients diagnosed with rectal or anal cancer after a prior diagnosis of prostate cancer from 1/1/1995 to 8/1/2019. From this list, we included the patients who have been previously treated with prostate external beam RT, brachytherapy, or a combination of both and then received a subsequent course of rectal or anal cancer RT (n=29). Three patients with anorectal melanoma or neuroendocrine tumors were then excluded. We only included patients with rectal adenocarcinoma or anal squamous cell carcinoma.

Data on patients and tumors general characteristics, treatment details including the use of a rectal spacer when applicable, outcomes, and late toxicity were collected from the electronic medical records. Toxicity data recorded in patient charts was graded per the Common Terminology Criteria for Adverse Events, version 5.0 (CTCAE) (7).

CT simulation scans with the corresponding generated RT treatment plans were extracted from the treatment planning system and a single dosimetrist (J.N.) contoured the bladder, bladder neck, rectum, and prostate. For palliative cases, the clinical target volume (CTV) is the gross tumor with a margin. For definitive cases, the CTV encompasses the perirectal, presacral, and internal iliac lymph nodes with or without the external iliac and inguinal nodes whenever possible as determined by the anatomy of the bowel in the radiation field and the doses received by small bowel during prior irradiation. We assessed the doses (maximum, minimum and mean) received by those organs at risk in addition to the coverage of gross and planning target volumes. For patients who received external beam treatment, we used the standard EQD2 formula: *EQD*2 = *D* *x* ([*d* + (*α*/*β*)]/[2 + (*α*/*β*)]) where D is the total dose to the organs at risk, in Gy and d is the dose/fraction in Gy. We used 3 as the α/β ratio. For the patients who received brachytherapy, we used the methodology outlined in a paper by Stock et al. (8). This summation methodology is only applicable to patients who received Low Dose Rate (LDR) brachytherapy. To summarize, it uses the summation of biologically effective dose (BED) calculations. For external beam radiation, *BED* = *nd*[1+(*d*/(*α*/*β*))], where n is the number of fractions and d is the dose per fraction. For Brachytherapy BED we slightly modified the equation from the paper. The paper used D90 as that paper was in reference to the prostate doses; in our case we used Dmax. The equation is as follows: *BED* = (*Dmax*){1+[Dmax* *λ*/(*μ*+ *λ*)(*α*/*β*)]}, where λ is a radioactive decay constant and μ is a repair constant. The BED was converted to EQD2 and summed.

Descriptive statistics were reported for patients’, tumors’, and treatment characteristics, toxicities, and dosimetry variables. Continuous variables were summarized in median and range and categorical variables were summarized in count and percent. The rate of development of late toxicities, including fistulae was assessed by sample proportion. The rate of acute toxicities and long-term toxicities associated with anorectal radiation therapy was summarized by toxicity grade and time after the radiation, respectively. The cumulative incidence of local and distant progression after anorectal radiation was estimated for anal and rectal cancer patients separately with death as a competing risk. Overall survival (OS) with the time between anorectal RT and death as the outcome of interest and progression free survival (PFS) with the time between end anorectal RT and death or progression of the disease as the outcome of interest were estimated for anal and rectal cancer patients. For patients with both local and distant progression, time to the first progression was calculated. All statistical analyses were performed using R statistical software, version 4.1.1 (https://www.r-project.org/).

## Results

A total of 26 patients were analyzed, 17 with rectal cancer and 9 with anal cancer. None had metastatic disease. All patients had a history of prior prostate RT. **Table 1** presents individual data on each of the 26 included patients. Median age at diagnosis of the prior prostate cancer was 69 (49, 79) years. Prior prostate RT was delivered using low dose rate brachytherapy (LDR) in 15/26 patients (57.7%) with a median dose of 145 Gy (144-160), external beam RT (EBRT) in 8 patients (30.8%) with 79.2Gy/44 or 81Gy/45 fractions (fr) as the most frequently used fractionations, and combination EBRT + LDR in 3 patients (11.5%) with 50.4/28 + 80-110 Gy I- 125 or Pd-103 LDR.

**Table 1:** Individual data on each of the twenty-six patients treated with radiation therapy for *de novo* rectal and anal cancers after prior prostate RT.

	Age at prior prostate cancer	Prostate RT modality	Prostate brachy dose (cGy)	Prostate EBRT dose (cGy)	Prostate EBRT fractions	Age at Dx of ano-rectal cancer	Location of ano-rectal tumor	Interval between prostate and ano-rectal RT (months)	Ano-rectal RT modality	Ano-rectal RT treatment group	Ano-rectal EBRT dose (cGy)	Ano-rectal EBRT fractions	Chemo concurrent 5FU rectal or 5FU-mitomycin C anal)	Clinical response on MRI and endoscopy	Ano-rectal cancer surgery	Acute side effects	Late side effects
**Rectal Cancer Patients**																	
**1**	69	LDR brachy				78	anterior	175	EBRT	BID	3900	26	yes	partial	no	fatigue	none
**2**	69	LDR brachy	16000			71	anterior	31	EBRT	Definitive EBRT	5040	28	yes	complete	yes	proctitis (grade 3)	none
**3**	58	EBRT +brachy	10800	4500	25	62	posterior	76	brachy	Palliative brachy			no		yes	none	none
**4**	54	LDR brachy	14500			66	posterior	147	EBRT	Definitive EBRT	4500	25	yes	partial	yes	cystitis (grade 3)	urinary cutaneous fistula and non-healing perineal wound
**5**	66	LDR brachy				91		303	EBRT	Definitive EBRT	5000	25	no	partial	no	fatigue and dermatitis (grade 3)	none
**6**	79	EBRT prostate		8100	45	91	posterior	149	brachy	Palliative brachy			yes	partial	no	fatigue and diarrhea	none
**7**	75	EBRT prostate	14500	7991	45	88	posterior	156	brachy	Palliative brachy			no	partial	no	none	none
**8**	74	LDR brachy				90	anterior	195	EBRT	BID	3000	20	yes	partial	no	fatigue and diarrhea	none
**9**	69	LDR brachy				82	anterior	166	EBRT	Definitive EBRT	5000	25	yes	complete	no	fatigue, diarrhea, and cystitis	none
**10**	71	EBRT prostate		7200	36	81	posterior	134	EBRT	Definitive EBRT	3000	5 *	no	stable disease	no	none	cystitis with hematuria
**11**	71	LDR brachy				86	lateral	195	EBRT	Definitive EBRT	4500	25	yes	complete	no	fatigue, proctitis, and dermatitis	none
**12**	71	LDR brachy	16000			80	anterior	182	EBRT	Definitive EBRT	5040	28	yes	complete	no	fatigue, diarrhea, and proctitis	none
**13**	68	LDR brachy	14400			87	anterior	236	EBRT	BID	3000	20	no	complete	no	fatigue	none
**14**	67	LDR brachy	16000			81	anterior	165	EBRT	Definitive EBRT	5040	28	yes	partial	yes	fatigue, diarrhea, and dermatitis	none
**15**	71	LDR brachy				80	anterior	97	EBRT	Definitive EBRT	5040	28	yes	partial	yes	fatigue, diarrhea, and proctitis	none
**16**	66	EBRT prostate		7560	42	73	posterior	80	EBRT	BID	3900	26	yes	partial	yes	fatigue, diarrhea, and proctitis	none
**17**	49	EBRT prostate		7920	44	52		36	brachy	Palliative brachy	1500		no		yes	none	none
**Anal Cancer Patients**																	
**1**	73	LDR brachy	14400			87		172	EBRT	Definitive EBRT	5000	25	yes	complete	no	fatigue, proctitis, and dermatitis	rectovesicular fistula
**2**	71	LDR brachy	14400			80	lateral	108	EBRT	Definitive EBRT	5000	25	yes	complete	no	fatigue, diarrhea, and proctitis	none
**3**	65	EBRT +brachy	8000	5040	28	73	posterior	104	EBRT	Definitive EBRT	5000	25	yes	complete	no	fatigue, diarrhea, and dermatitis	stool incontinence
**4**	64	LDR brachy	14400			78	lateral	162	EBRT	Definitive EBRT	5000	25	yes	complete	no	fatigue, proctitis, and dermatitis	none
**5**	68	EBRT +brachy	11000	5040	28	75	anterior	99	EBRT	Definitive EBRT	5000	25	yes	complete	no	fatigue and dermatitis	none
**6**	64	LDR brachy				75	posterior	149	EBRT	Definitive EBRT	4800	24	yes	complete	no	diarrhea and proctitis	none
**7**	78	EBRT prostate		8639.99	48	81	lateral	42	EBRT	BID	3750	25	no	partial	no	fatigue, proctitis, and dermatitis	none
**8**	62	EBRT prostate		6840	38	79	posterior	129	EBRT + brachy	EBRT + brachy	2520	20	yes	partial	no	fatigue and proctitis	none
**9**	72	EBRT prostate		7920	44	72	posterior	6	EBRT + brachy	EBRT + brachy	5000	25	yes	partial	no	fatigue and dermatitis	none

RT, Radiation therapy; EBRT, External beam radiation therapy; chemo, chemotherapy; brachy, brachytherapy; LDR, low dose rate; BID, twice daily. * this patient received two courses of 3000cGy in 5 fractions.

Median age for development of anorectal cancer was 80 (52, 91) years. Most patients (73%) had an excellent performance status (KPS: 80-100). Median interval between prostate and anorectal RT was 12.3 years (0.5 - 25.3). Patients, tumor, and treatment characteristics for anorectal cancers are summarized in **Table 2**. Most patients with rectal and anal cancers presented with stage II disease, 41% and 44% respectively. 53% of rectal cancer tumors and 25% of anal cancer tumors were located anteriorly. RT for rectal cancer was delivered most commonly with 50.4Gy/28 fr or 1.5 Gy twice daily fractionation to 30-45 Gy. The most used RT dose for anal cancer was 50Gy/25 fr. Endorectal high dose rate (HDR) brachytherapy was used in 6 (21%) patients: as a boost after EBRT in 2 patients with anal cancer and alone in 4 rectal cancer patients. Treatment was delivered with definitive EBRT doses, combination of EBRT and brachytherapy, BID fractionation (1.5 Gy/fraction), and palliative endorectal HDR brachytherapy in 15 (57.7%), 2 (7.7%), 5 (19.2%), and 4 (15.4%) patients, respectively. 65% and 89% of rectal and anal cancer patients got concurrent chemotherapy, respectively. Seven patients with rectal cancer (41%) underwent surgery after the second course of radiation therapy, revealing pathological stage II disease in 56.8%. Five patients with rectal cancer had complete clinical response, four of which did not undergo surgery. Out of the ten rectal cancer patients who didn’t undergo surgical resection, five had comorbidities and weren’t surgical candidates, four refused surgery and one patient had extensive disease where surgery was thought to be non-curative.

**Table 2:** Tumor characteristics, treatment characteristics, and response for patients treated with radiation therapy for *de novo* rectal and anal cancers after prior prostate RT.

Characteristics				Rectal cancer* (n=17) frequency (%)/median (range)	Anal cancer (n=9) frequency (%)/median (range)
AJCC Stage	Stage I			2 (11.76%)	0
	Stage IIA			6 (35.29%)	3 (33.33%)
	Stage IIB			0	1 (11.11%)
	Stage IIC			1 (5.88%)	NA
	Stage IIIA			0	2 (22.22%)
	Stage IIIB			2 (11.76%)	2 (22.22%)
	Stage IIIC			3 (17.65%)	1 (11.1%)
	Recurrent			3 (17.65%)	0
Size (MRI) in cm				3.2 (1.5, 8.0)	3 (1.2, 6.5)
Location	anterior			8 (53.33%)	1 (12.5%)
	posterior			6 (40%)	4 (50%)
	lateral			1 (6.67%)	3 (37.5%)
Anal sphincter involvement				5 (33.33%)	7 (77.78%)
Prostate/bladder/SV or levator ani involvement (T4)				5 (31.25%)	1 (11.11%)
RT modality	EBRT			13 (76.47%)	9 (100%)
	Brachytherapy alone			4 (23.53%)	0
	Brachytherapy boost after EBRT			0	2 (22.22%)
RT treatment group		Definitive EBRT		9 (52.94%)	6 (66.67%)
		Palliative		4 (23.53%)	0
		Combo brachy +EBRT		0	2 (22.22%)
		BID		4 (23.53%)	1 (11.11%)
RT brachytherapy dose (cGy)		500/1		1 (25%)	0
		1200-1500/3		1 (25%)	1 (50%)
		1500-1750/1		2 (50%)	0
		1950/3		0	1 (50%)
RT EBRT dose (cGy)				4500 (3000, 5040)	5000 (2520,5000)
RT EBRT fractions				25 (5, 28)	25 (20, 25)
Two courses of anorectal RT after prior prostate RT				1 (5.88%) with a dose of 3000 cGy/5 fractions given twice	0
Chemotherapy concurrent (5FU based)				11 (64.71%)	8 (88.89%)
Chemotherapy adjuvant/neoadjuvant				10 (58.82%) (70% FOLFOX and 30% 5FU)	2 (22.22%) (50% MMC+5FU and 50% Cisplatin+5FU
Clinical response			stable	1 (6.7%)	0
			partial response	9 (60%)	3 (33.33%)
			complete response	5 (33.33%)	6 (66.7%)
			missing	2	0
Pathological response: pTN			I	1 (14.29%)	NA
			IIA	3 (42.86%)	NA
			IIB	1 (14.29%)	NA
			IIIB	1 (14.29%)	NA
			IIIC	1 (14.29%)	NA
LVI positive				3 (50%)	NA
Rectal spacer				0	1 (11.11%)

*: median distance of 4.5 cm from the anal verge.

SV, seminal vesicles; RT, Radiation therapy; EBRT, External beam radiation therapy.

One patient with anal cancer (11%) underwent rectal spacer placement prior to the second course of definitive EBRT radiation. This patient had no acute cystitis during treatment and didn’t develop fistula or other long term side effects with a follow up of 22 months.

**Table 3** summarizes acute toxicities associated with anorectal radiation after prior prostate RT. Fatigue was the most common acute toxicity (73%), followed by proctitis (42%), and diarrhea (38%). Most toxicities were Grades 1-2. Grade 3 dermatitis, cystitis, and proctitis occurred in one, one, and one patient respectively. No Grade 4-5 acute toxicities were were reported. Long-term toxicity rates are summarized in **Table 4**. Two patients developed fistulae, one was urinary-cutaneous after prostate LDR and 45Gy/25fr for rectal cancer, and the other recto-vesicular after prostate LDR and 50Gy/25fr for anal cancer. The patient who developed the urinary-cutaneous fistula also developed a perineal abscess. Both fistulae were repaired by extensive surgeries; cystoprostatectomy and ileal conduit in one patient and a diverting colostomy in the other. There was no evidence of local recurrence at the time of development of fistula. Both patients had more than 10 years between the two RT courses.

**Table 3:** Acute toxicities associated with anorectal radiation therapy (RT) after prior prostate RT.

Acute Toxicities (N=26)	n (%)	Grade: n (%)	
		Gr 1-2	Gr 3*
**Fatigue**	19 (73)	19 (100)	0
**Diarrhea**	10 (38)	10 (100)	0
**Proctitis**	11 (42)	10 (91)	1 (9)
**Dermatitis**	9 (35)	8 (89)	1 (11)
**Cystitis**	2 (8)	1 (50)	1 (50)

* No Gr 4+ toxicity.

Gr, Grade.

**Table:** Table 4 The rate of developing long-term toxicities associated with anorectal radiation therapy (RT) after prior prostate RT.

Long-term Toxicities		Rectal cancer (n=17)	Anal Cancer (n=9)
**n (%) and time after RT**			
**Fistula**	1 (6%) at 30 mo		1 (11%) at 7 mo
**Others: hematuria, perineal abscess, or fecal incontinence**	2 (12%) at 15 and 45 mo (hematuria and perineal abscess)		1 (fecal incontinence) (11%) at 77 mo

mo, months.

In 11 patients with available dosimetry, coverage was adequate with a median GTV V100% of 100% (68.1%-100%) and PTV V100% of 97.5% (89.6%-100%). In the patient with available EQD2 cumulative doses, median EQD2 cumulative dose to the rectum and bladder were 11764 cGy and 11540 cGy respectively. Correlations between dosimetry and toxicity could not be accurately performed due to the small sample size with few numbers of events.

**Figure 1:**
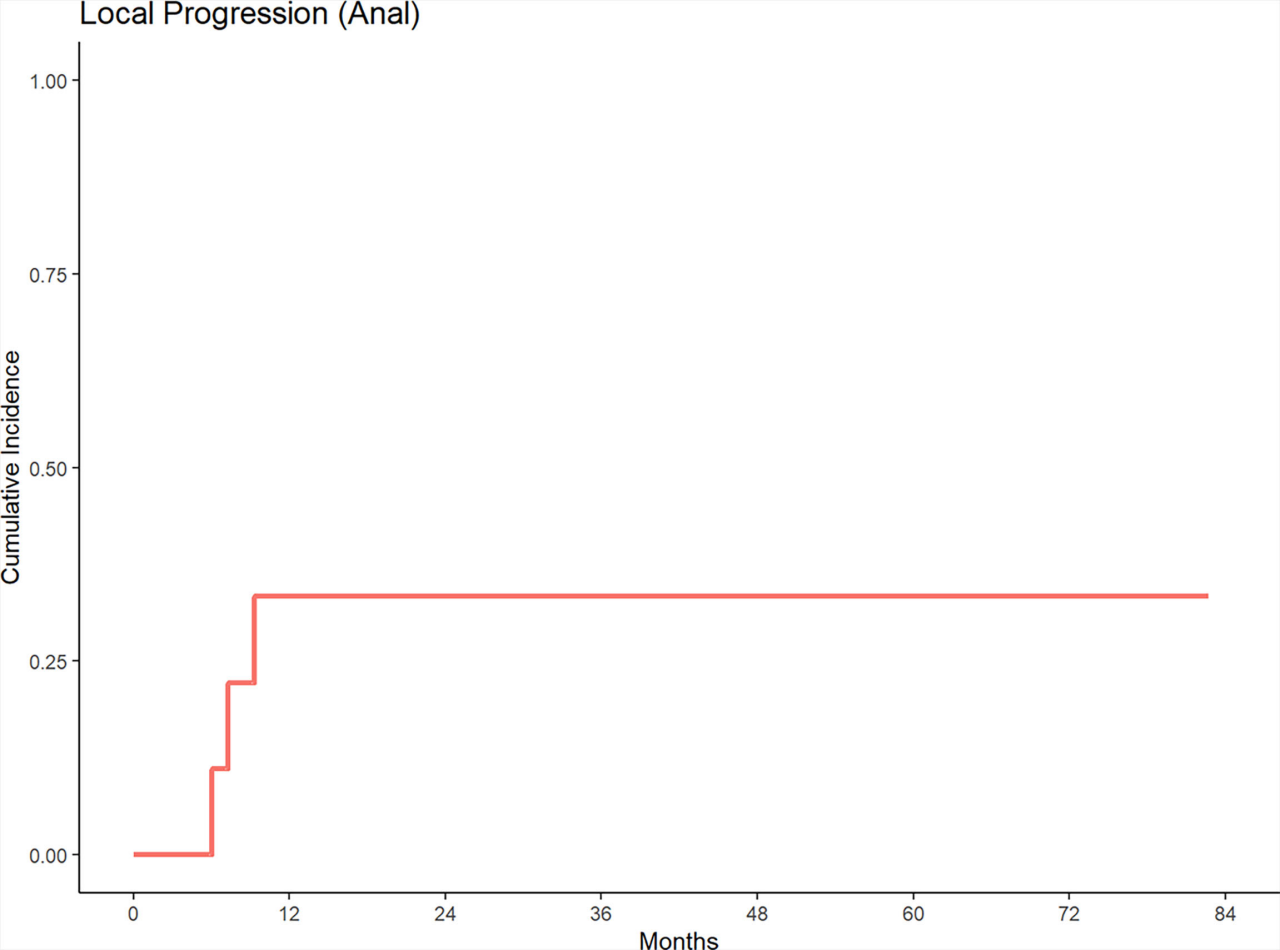
Cumulative incidence of local progression for patients treated with a second course of pelvic radiation for *de novo* anal cancers after prior prostate radiation.

**Figure 2:**
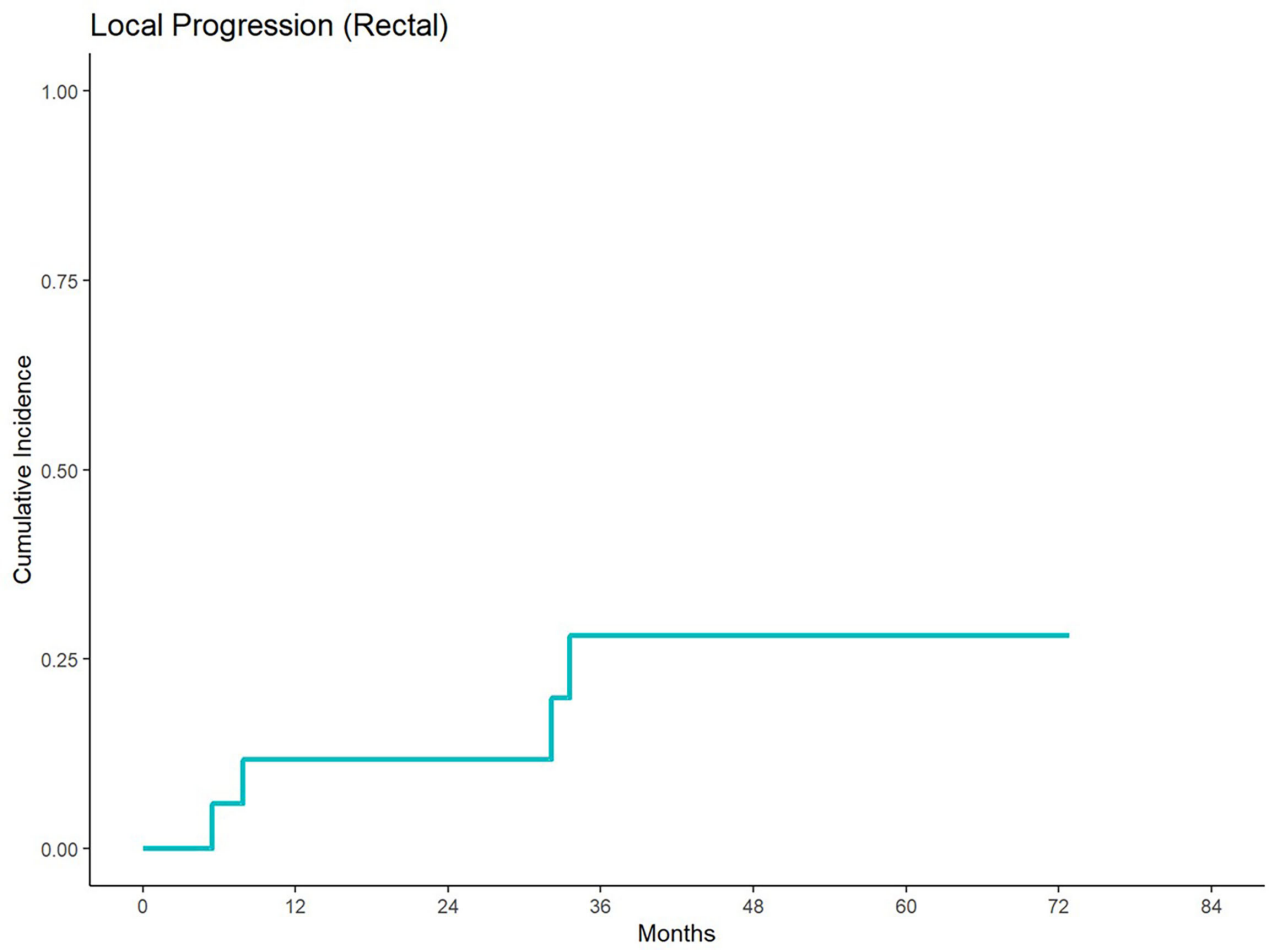
Cumulative incidence of local progression for patients treated with a second course of pelvic radiation for *de novo* rectal cancers after prior prostate radiation.

With a median follow up of 84.42 months, 5-year cumulative incidence of local progression was 35% and 30% for anal and rectal cancers, respectively (**Figures 1**, **2**). Out of 15 patients who received definitive EBRT doses, two patients had local progression. The other 5 local progressions in our patient cohort were in patients who received palliative brachytherapy (one patient), combination EBRT and brachytherapy (two patients), and BID EBRT (two patients).

**Figure 3:**
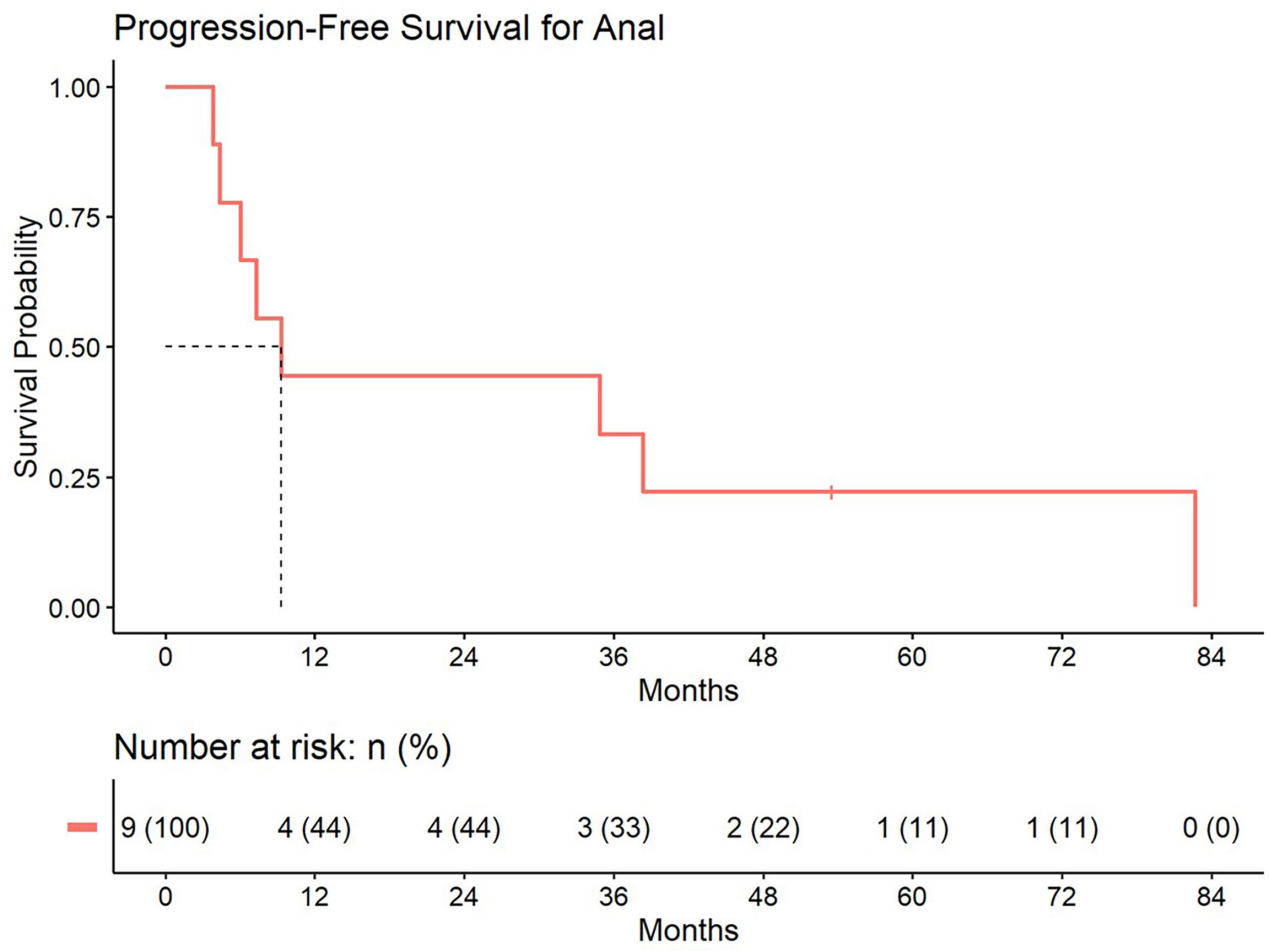
Progression free survival for patients treated with a second course of pelvic radiation for *de novo* anal cancers after prior prostate radiation.

**Figure 4:**
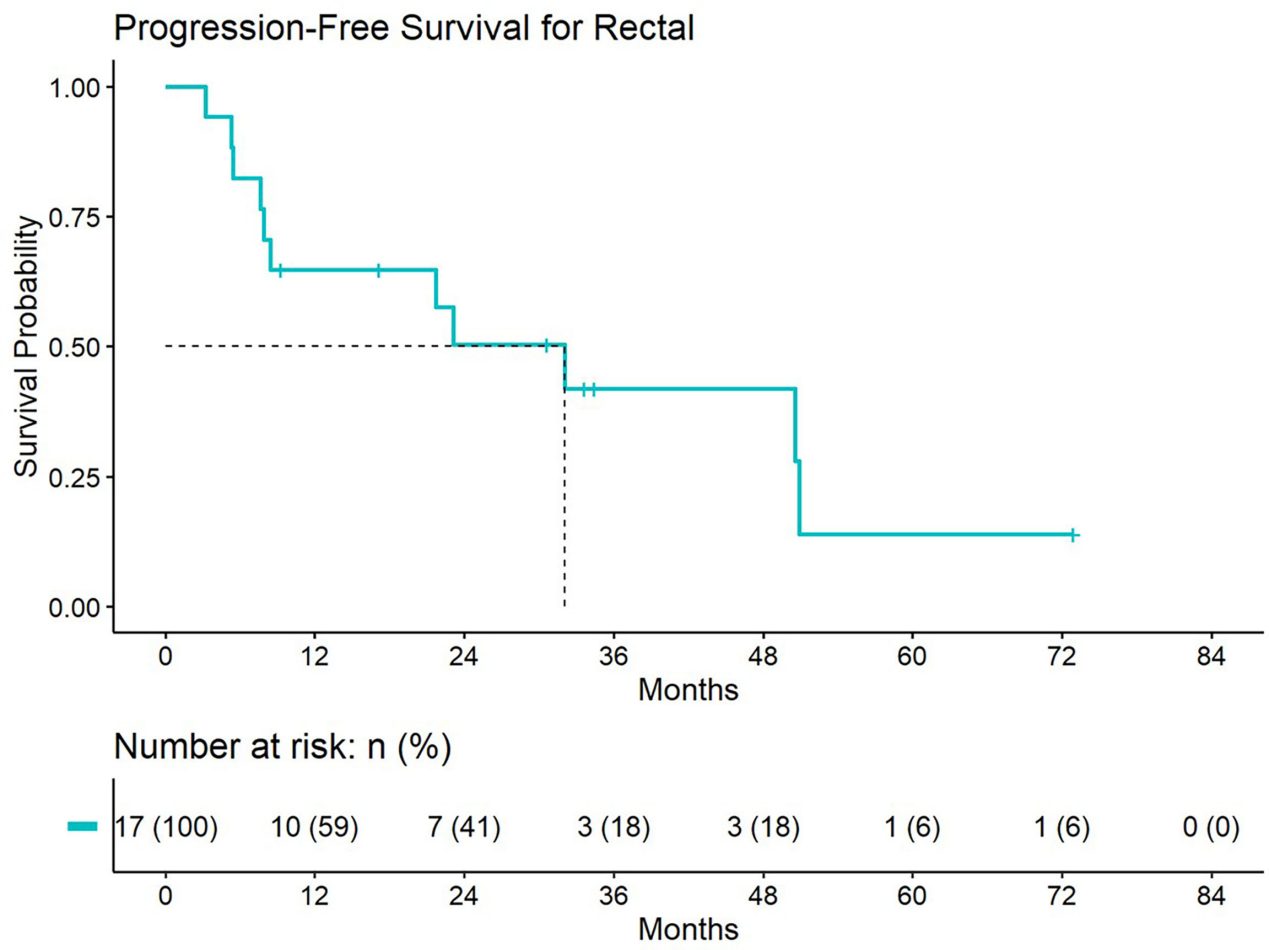
Progression free survival for patients treated with a second course of pelvic radiation for *de novo* rectal cancers after prior prostate radiation.

**Figure 5:**
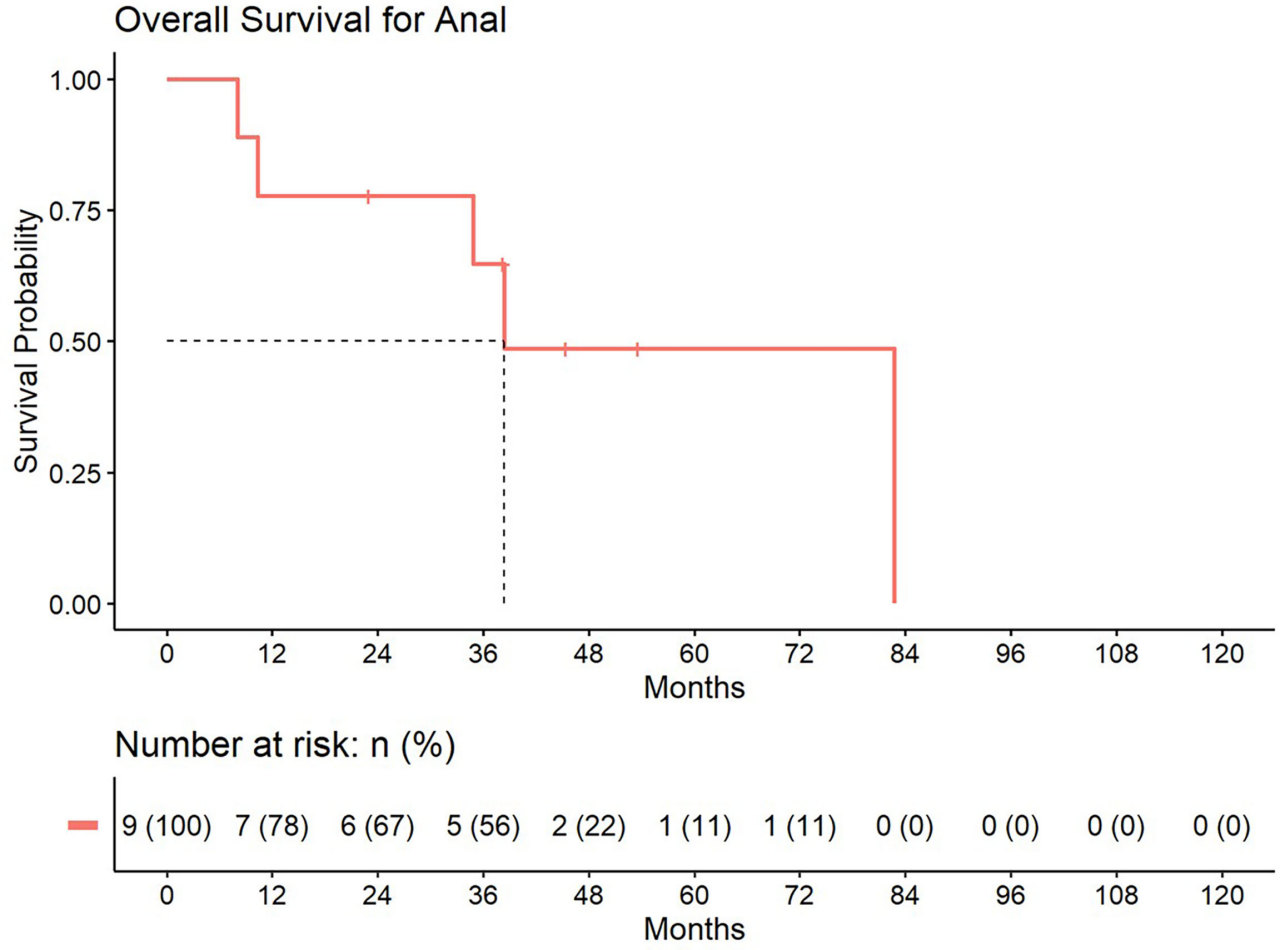
Overall survival for patients treated with a second course of pelvic radiation for *de novo* anal cancers after prior prostate radiation.

**Figure 6:**
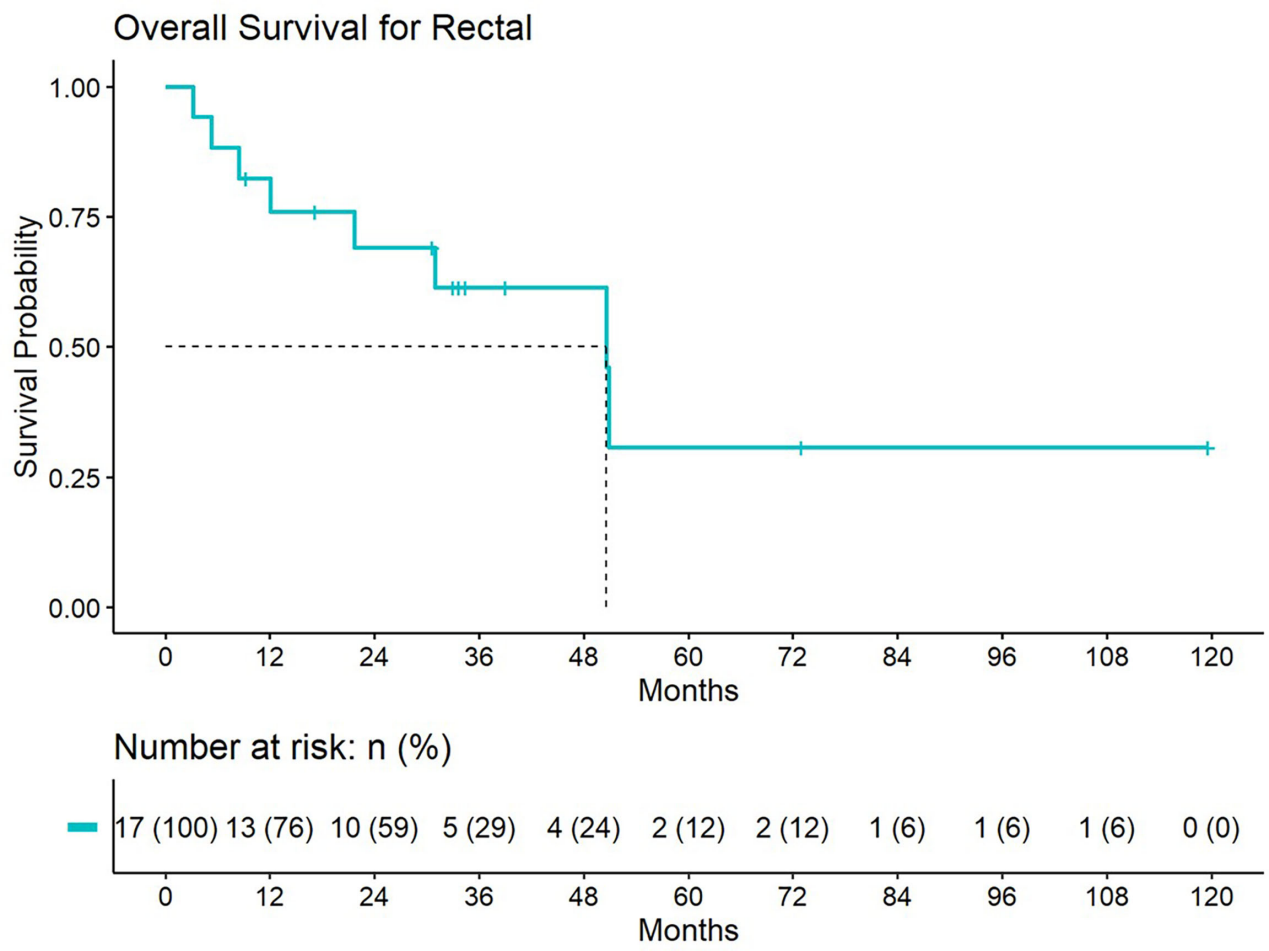
Overall survival for patients treated with a second course of pelvic radiation for *de novo* rectal cancers after prior prostate radiation.

5-year distant progression rate was around 23% and 19% for anal and rectal cancers respectively. The median progression free survival for rectal and anal cancer patients was 32.1 months and 9.3 months, respectively. 2-year PFS was 44.4% and 50% for anal and rectal cancers, respectively (**Figures 3**, **4**). Median OS and 5-year OS were 38.4 months and 49% for anal cancer and 50.6 months and 31% for rectal cancer, respectively (**Figures 5**, **6**).

## Discussion

Our series describing RT for *de novo* anorectal cancers after prior prostate cancer irradiation tackles a challenging clinical scenario. With patients surviving longer than 5-10 years after their first radiation treatment (9), a second course of treatment for a secondary malignancy is becoming more common (10). Radiation therapy has been usually avoided in such scenarios given concern for potential complications. In our series, RT for anorectal cancers after prior prostate irradiation was feasible with local control of around 65-70% at 5 years. Of note is that 10 out of 19 patients with rectal cancers in our cohort did not undergo surgical resection due to comorbidities, refusal, or extensive disease. Also, four rectal cancer patients received palliative endorectal brachytherapy. It’s important to also note that the 5-yr OS of 49% and 31% for anal and rectal cancer patients, respectively, is probably affected by the older population (median age of 80 years). Toxicity was also acceptable with three patients (11.5%) developing grade 3 acute toxicity and four patients (15%) developing grades 2-4 late toxicity, including the two patients who developed fistulae. In this population of patients whose therapy options are limited by prior radiation, local control achieved with the second course of radiation was good, especially in patients who received definitive EBRT doses (45-50Gy) in our series.

Pelvic reirradiation has been reported in the setting of recurrent rectal cancers. In a study from MDACC on 102 patients, reirradiation for recurrent rectal cancers with a median dose of 39 Gy in 26 fractions using 1.5 Gy BID fractionation resulted in 3-year freedom from local progression of 49% in patients undergoing surgery and 30% in those not undergoing surgery. The 3-year rate of Grades 3-4 toxicities in their study was 34% and two patients were reported to develop fistulae (4). Their reported rate of local control is lower than the rate in our series, likely given the usual worse prognosis of recurrent compared to primary rectal tumors. Another more recently published study from MDACC included a small series of 10 patients who had *de novo* rectal cancer, similar to our cohort, but were all treated with hyperfractionated accelerated radiation therapy after a prior radiation course for different pelvic malignancies including bladder, prostate, and gynecologic malignancies. The 3-year rate of freedom from local progression was 62%, comparable to the findings of our study. The toxicity in their study was low with one acute and one late grade 3 toxicity. The duration between the two RT courses was long with a median of 15 years, comparable to the median duration of around 12 years in our cohort, which probably contributed to the relatively low rate of grade 3+ toxicities (11).

Although toxicity was relatively acceptable in our cohort of patients, it remains a major concern for patients who undergo a second course of pelvic radiation, especially after prior prostate LDR brachytherapy. A prostate implant might deliver a high dose to the rectum and such patients should be treated with extra caution if a second course of radiation is to be delivered. Although we didn’t have reported cases of rectal bleeding in our cohort, it’s also one of the possible side effects that should be discussed when offering a second course of pelvic radiation. Our two patients who developed fistulae both required extensive surgeries. This raises the question on ways to decrease the risk of fistulae in such a patient population. The two patients who developed fistulae both received conventional fractionation for their second course of pelvic irradiation and prior LDR brachytherapy for prostate cancer. The sample size is too small to derive valid conclusions on whether the use of different fractionation schedules, such as hyperfractionated RT, might have decreased this risk. We could not retrieve the cumulative doses to the rectum using the old brachytherapy plans for the two patients who developed fistulae in our cohort but the dose to the rectum in such cases is important to take into consideration when deciding on the feasibility of a second course of radiation.

Another potential strategy for decreasing the risk of fistulae could possibly be by rectal spacer placement. The use of a rectal spacer to increase the separation between the rectum and the urinary tract in patients undergoing prostate cancer radiation therapy is now FDA approved and has been shown in several studies to decrease rectal toxicity (12). One of the most widely studied of these spacer materials is a polyethylene glycol hydrogel that is injected as a liquid that expands the perirectal space by solidifying into a soft absorbable spacer. The spacer has been shown to be stable during the duration of a typical radiation therapy course (13). The insertion of a rectal spacer for re-irradiation has also been reported in the setting of locally recurrent gynecologic (GYN) malignancies. In a study from Brigham and Woman’s hospital, three patients with locally recurrent GYN malignancies requiring reirradiation underwent spacer insertion between the rectum and the vagina. The average dose to the rectum was reduced by 11% and to the sigmoid by 45% (14). There is no data however on its use to decrease urinary toxicity in patients undergoing rectal or anal cancer radiation after prior prostate radiation therapy. The aim of the spacer in such cases would be to minimize dose to the urinary tract (prostate and bladder) and would be appropriate only for anorectal tumors that are located posteriorly. Inserting a rectoprostatic spacer is more challenging after prior radiation to the pelvis but is feasible in some patients. In a study on 11 patients with prostate cancer and prior radiotherapy, spacing was achieved successfully in 8 out of the 11 (73%) but was not possible in 3 patients due to fibrosis (15). The feasibility and safety of using a hydrogel spacer prior to radiation therapy in patients with posteriorly located anorectal cancers and a history of prior prostate radiation therapy is currently being investigated at our institution.

Proton therapy represents a second promising alternative to photon radiation to reduce toxicity in the setting of a second course of pelvic RT especially in select situations where patients’ anatomy is limited by adjacent organs at risk, mainly the urinary tract. A study by Koroulakis et al. on 28 patients who received a second course of pelvic proton radiation therapy with a median dose of 44 Gy, delivered mostly twice daily, included 10 patients treated for *de novo* rectal cancer. Three patients out of the whole cohort (10.7%) developed grade 3 acute toxicities, and 1 did not complete the second course of radiation owing to toxicity. Four (14.2%) experienced late grade<3 toxicity, including 1 patient who had a grade 5 toxicity. The patient who developed grade 5 toxicity had a history of late toxicity from prior whole abdominal and pelvic boost RT for ovarian cancer in the 1970s, that included late radiation colitis, cystitis, and loss of a kidney. After the second course of RT, she developed rectal bleeding, rectovaginal fistula and ultimately a bleeding stage IV ulcer and died of presacral hemorrhage at 26 months. The 1-year local progression rate was 33.7% and 1-year OS was 81.8% (16). Besides proton therapy, other high-tech modalities such as magnetic resonance images (MRI) Linacs, especially with adaptive planning might also be promising by allowing more precise targeting of the anorectal gross tumor volume (GTV). This allows for smaller radiation fields which would possibly minimize dose to adjacent organs at risk such as the bladder and the prostate.

In addition to the inherent limitations of a retrospective study, our sample size with a relatively small number of events is a main limitation that prevented us from investigating accurate correlation measures between dosimetry variables and toxicities and local control. We were also not able to compare outcomes among rectal cancer patients who underwent curative surgery and those who did not due to the small number of patients. Our cohort of patients were treated with different RT regimens that included both conventionally fractionated and hyperfractionated courses, including brachytherapy, once-daily EBRT, twice-daily EBRT, and combination EBRT and brachytherapy. Comparison among the regimens was not feasible with our sample size. Also, it’s hard to tell whether some of the anorectal cancers, especially the anteriorly located ones, are radiation induced malignancies. Another limitation is that the assessment of late toxicities was extracted from the patients’ medical records and thus was not standardized. Nevertheless, data on radiation therapy for *de novo* anorectal cancers after prior prostate RT is scarce. Thus, our study can provide insight into the feasibility of a second course of pelvic RT in such a patient population whose treatment options are limited and where the morbidity of a local recurrence can significantly affect quality of life.

Further studies with larger sample size are needed to assess prognostic variables for local control and toxicities and to investigate strategies that aim to improve outcomes and minimize radiation induced toxicities, especially in patients with prior LDR brachytherapy implants.

## Conclusion

Radiation therapy for *de novo* anorectal cancers after prior prostate cancer RT is feasible with local control of 65-70% at 5 years but should be delivered with caution since it poses a risk of fistulae and possibly bleeding, especially in patients treated with prior LDR brachytherapy. Further larger studies, perhaps using proton therapy and/or rectal hydrogel spacers, are needed to further establish the safety of RT in this context, decrease toxicity, and improve outcomes.

## Data availability statement

The raw data supporting the conclusions of this article will be made available by the authors, without undue reservation.

## Ethics statement

The studies involving human participants were reviewed and approved by Memorial Sloan Kettering Cancer Center Institutional Review board (MSKCC IRB 16- 370). Written informed consent for participation was not required for this study in accordance with the national legislation and the institutional requirements.

## Author contributions

LH collected data and wrote the manuscript. AW contributed to conceptualizing the work and reviewing and editing. JN and SB worked on the dosimetry part of the paper. SY, ZZ, and XB did the data analysis and reviewed the paper. MR, JC, AD, PR, MZ, MN, EP, MJZ, and CC reviewed and edited the paper. CH conceptualized the work, edited and supervised the writing. All authors contributed to the article and approved the submitted version.

## Acknowledgments

This work has been presented as a poster presentation at the American Society of Therapeutic Radiation Oncology (ASTRO) 2021 Annual Meeting.

## Conflict of interest

PR reports prior research funding from and is a consultant for EMD Serono. MJZ is a consultant for Boston Scientific. 

The remaining authors declare that the research was conducted in the absence of any commercial or financial relationships that could be construed as a potential conflict of interest.

## Publisher’s note

All claims expressed in this article are solely those of the authors and do not necessarily represent those of their affiliated organizations, or those of the publisher, the editors and the reviewers. Any product that may be evaluated in this article, or claim that may be made by its manufacturer, is not guaranteed or endorsed by the publisher.

## References

[1] Baxter NN Tepper JE Durham SB Rothenberger DA Virnig BA. Increased risk of rectal cancer after prostate radiation: A population-based study. Gastroenterology (2005) 128(4):819–24. doi: 10.1053/j.gastro.2004.12.038 15825064

[2] NCCN. Rectal cancer. In: Rectal cancer (2020). Available at: https://www.nccn.org/professionals/physician_gls/pdf/rectal.pdf.

[3] NCCN. Anal cancer. In: Anal cancer (2020). Available at: https://www.nccn.org/professionals/physician_gls/pdf/anal.pdf.

[4] Das P Delclos ME Skibber JM Rodriguez-Bigas MA Feig BW Chang GJ. Hyperfractionated accelerated radiotherapy for rectal cancer in patients with prior pelvic irradiation. Int J Radiat Oncol [Internet]. (2010) 77(1):60–5. http://www.sciencedirect.com/science/article/pii/S0360301609006555. doi: 10.1016/j.ijrobp.2009.04.056 19695792

[5] Osborne EM Eng C Skibber JM Rodriguez-Bigas MA Chang GJ Nancy You Y-Q. Hyperfractionated accelerated reirradiation for patients with recurrent anal cancer previously treated with definitive chemoradiation. Am J Clin Oncol (2018) 41(7):632–7. doi: 10.1097/COC.0000000000000338 27755060

[6] Chang DT Koay EJ Herman JM Hong TS Das P. Abdominal and pelvic reirradiation for recurrent gastrointestinal cancers. Semin Radiat Oncol (. 2020) i:232–7. doi: 10.1016/j.semradonc.2020.02.006 32503788

[7] Cancer Therapy Evaluation Program (CTEP). Common terminology criteria for adverse events (CTCAE).v.5.0 [5x7]. In: Cancer ther eval progr, vol. 155. (2017). Available at: https://ctep.cancer.gov/protocolDevelopment/electronic_applications/ctc.htm#ctc_50.

[8] Stock RG Stone NN Cesaretti JA Rosenstein BS. Biologically effective dose values for prostate brachytherapy: Effects on PSA failure and posttreatment biopsy results. Int J Radiat Oncol [Internet]. (2006) 64(2):527–33. https://www.sciencedirect.com/science/article/pii/S0360301605023722. doi: 10.1016/j.ijrobp.2005.07.981 16242258

[9] Hall MD Schultheiss TE Smith DD Tseng BP Wong JYC. The impact of increasing dose on overall survival in prostate cancer. Radiat Oncol (2015) 10:115. doi: 10.1186/s13014-015-0419-3 25990489 PMC4448310

[10] Morton LM Onel K Curtis RE Hungate EA Armstrong GT. The rising incidence of second cancers: patterns of occurrence and identification of risk factors for children and adults. Am Soc Clin Oncol Educ book (2014) e57–67. doi: 10.14694/EdBook_AM.2014.34.e57 24857148

[11] Jensen G Tao R Eng C Skibber JM Rodriguez-bigas M Chang GJ. Treatment of primary rectal adenocarcinoma after prior pelvic radiation : The role of hyperfractionated accelerated reirradiation. Advancesradonc (2018) 3(4):595–600. doi: 10.1016/j.adro.2018.07.003 PMC6200883 30370360

[12] Mariados N Sylvester J Shah D Karsh L Hudes R Beyer D. Hydrogel spacer prospective multicenter randomized controlled pivotal trial: Dosimetric and clinical effects of perirectal spacer application in men undergoing prostate image guided intensity modulated radiation therapy. Int J Radiat Oncol Biol Phys [Internet]. (2015) 92(5):971–7. doi: 10.1016/j.ijrobp.2015.04.030 26054865

[13] Uhl M Herfarth K Eble MJ Pinkawa M van Triest B Kalisvaart R. Absorbable hydrogel spacer use in men undergoing prostate cancer radiotherapy: 12 month toxicity and proctoscopy results of a prospective multicenter phase II trial. Radiat Oncol (2014) 9(1):96. doi: 10.1186/1748-717X-9-96 24758224 PMC4016630

[14] Viswanathan AN Damato AL Nguyen PL. Novel use of a hydrogel spacer permits reirradiation in otherwise incurable recurrent gynecologic cancers. J Clin Oncol [Internet]. 2013/10/21. (2013) 31(34):e446–7. doi: 10.1200/JCO.2012.47.9931 PMC5795666 24145342

[15] Mahal BA Ziehr DR Hyatt AS Neubauer-Sugar EH O’Farrell DA O’Leary MP. Use of a rectal spacer with low-dose-rate brachytherapy for treatment of prostate cancer in previously irradiated patients: Initial experience and short-term results. Brachyther (2014) 13(5):442–9. doi: 10.1016/j.brachy.2014.05.001 24880584

[16] Koroulakis A Molitoris J Kaiser A Hanna N Bafford A Jiang Y. Reirradiation for rectal cancer using pencil beam scanning proton Therapy : A single institutional experience. Advancesradonc [Internet]. (2021) 6(1):100595. doi: 10.1016/j.adro.2020.10.008 PMC7807140 33490730

